# Enablers and barriers to effective parenting within the first 1000 days of life

**DOI:** 10.1093/eurpub/ckac131.452

**Published:** 2022-10-25

**Authors:** B Adebiyi, T Goldschmidt, F Benjamin, I Sonn, E Rich, N Roman

**Affiliations:** Centre for Interdisciplinary Studies of Children, Families and Society, University of the Western Cape, Cape Town, South Africa

## Abstract

**Background:**

The first 1000 days is the period between conception and a child’s second birthday. Globally, research on parenting is in an advanced stage, but parenting research focusing specifically on parenting in this developmental phase is limited in South Africa. Therefore, this study explores the enablers and barriers to effective parenting within the first 1000 days through the lens of parents and caregivers in low socio-economic communities.

**Methods:**

This study was conducted in low socio-economic communities of South Africa. An exploratory qualitative research design explored the enablers and barriers to effective parenting within the first 1000 days of life. Thirty participants were purposively selected and interviewed in this study. A semi-structured interview schedule was used for all interviews. The data were analysed using inductive thematic analysis.

**Results:**

Two main categories emerged (effective parenting enablers and effective parenting barriers) during the data analysis. The main enablers of effective parenting within the first 1000 days of life include a support system, healthy behaviours/environment, job opportunities, religion, information/knowledge, and professional assistance. On the other hand, the main barriers to effective parenting were low socio-economic circumstances, environmental circumstances, lack of partner’s support, the negative impact of technology, and lack of access to services.

**Conclusions:**

Enablers that need to be promoted for effective parenting range from support systems to professional assistance for parents. Also, barriers that need to be removed for effective parenting range from low socio-economic circumstances to a lack of partner’s support for parents. This is because effective parenting is vital in improving developmental outcomes for children within the first 1000 days of life. Therefore, there is a need to develop policies and interventions to promote effective parenting within the first 1000 days in the communities.

**Key messages:**

• Effective parenting is vital in improving developmental outcomes for children within the first 1000 days of life.

• There is a need to develop policies and interventions to promote effective parenting within the first 1000 days in low socio-economic communities.

